# Multi-platform profiling of over 2000 sarcomas: Identification of biomarkers and novel therapeutic targets

**DOI:** 10.18632/oncotarget.3498

**Published:** 2015-03-26

**Authors:** Sujana Movva, Wenhsiang Wen, Wangjuh Chen, Sherri Z. Millis, Zoran Gatalica, Sandeep Reddy, Margaret von Mehren, Brian A. Van Tine

**Affiliations:** ^1^ Fox Chase Cancer Center, Philadelphia, PA, USA; ^2^ Caris Life Sciences, Phoenix, AZ, USA; ^3^ Washington University in St. Louis, St. Louis, MO, USA

**Keywords:** sarcoma, biomarkers, targeted therapies, DNA sequencing, protein expression

## Abstract

Background: Drug development in sarcoma has been hampered by the rarity and heterogeneity of the disease and lack of predictive biomarkers to therapies. We assessed protein expression and gene alterations in a large number of bone and soft tissue sarcomas in order to categorize the molecular alterations, identify predictive biomarkers and discover new therapeutic targets. Methods: Data from sarcoma specimens profiled for protein expression, gene amplification/translocation and DNA sequencing was reviewed. Results: 2539 sarcoma specimens of 22 subtypes were included. TOPO2A was the most overexpressed protein at 52.8%. There was overexpression or loss of other sarcoma relevant proteins such as SPARC, PTEN and MGMT. Approximately 50% of the sarcomas expressed PD-L1 by IHC and presented with PD-1+ TILs, notably the LMS, chondrosarcomas, liposarcomas and UPS. Gene amplification/rearrangement of *ALK, cMYC, HER2, PIK3CA, TOPO2A* and *cMET* was relatively uncommon. *EGFR* gene amplification occurred at a rate of 16.9%. DNA sequencing of 47 genes identified mutations in 47% of the samples. The most commonly mutated genes were *TP53* (26.3%) and *BRCA2* (17.6%). Overexpression of TOPO2A was associated with *TP53* mutation (*P* = 0.0001). Conclusion: This data provides the landscape of alterations in sarcoma. Future clinical trials are needed to validate these targets.

## INTRODUCTION

Sarcomas are a rare, heterogeneous group of mesenchymal tumors. With over 100 subtypes, a single therapeutic strategy for this group of cancers is unfeasible. For patients with advanced disease, the selection of therapy is based on the specific sarcoma subtype as well as the patient's fitness to receive aggressive chemotherapy. With this approach, median overall survival for patients with advanced bone and soft tissue sarcomas (STS) remains under two years [1]. Sarcomas can be classified according to the genetic alterations involved in their development: those with oncogenic somatic mutations [e.g. gastrointestinal stromal tumors (GIST)], those with DNA copy number alterations (e.g. dedifferentiated liposarcomas), and those with recurrent chromosomal translocations resulting in abnormal fusion proteins (e.g. synovial sarcomas). More commonly, sarcomagenesis is a result of complex chromosomal abnormalities, as in the case of leiomyosarcomas and high grade undifferentiated pleomorphic sarcomas [2].

Analyses of genetic alterations in sarcoma have generally focused on particular subtypes [3, 4] and frequently employ limited methodologies. Data from large scale, systematic genomic profiling of sarcomas is limited. Barretina and colleagues characterized 207 sarcoma specimens, including seven different subtypes, by DNA sequence, copy number alterations and mRNA expression. They were able to identify potentially druggable PIK3CA mutations in 18% of myxoid liposarcomas [5]. Conversely, a separate series, by Cote and colleagues found that when solely using DNA hotspot analysis or whole exome sequencing, mutations were rare and there was no pattern of alterations noted within sarcoma subtypes [6].

Protein biomarkers have been studied for many decades, in an attempt to predict which tumors will respond to what therapies. This is best highlighted in the breast cancer field, where the proteins estrogen receptor (ER), progesterone receptor (PR), and human epidermal growth factor receptor (HER2) have been well established as prospective treatment response biomarkers [7, 8]. To date, a large analysis for common chemotherapy associated biomarkers has not been performed across a large group of sarcomas. A detailed mapping of the therapy associated biomarkers will allow for identification of both targets and histologies that may respond to a particular drug, in order to design better clinical trials [9].

Using a registry of 2539 patients with bone and soft tissue sarcomas we have catalogued changes in protein expression, gene amplification/translocation, and somatic mutations. Herein we describe these findings with the goal of systematically characterizing the molecular alterations in a variety of sarcoma subtypes, identifying potential biomarkers of sensitivity to chemotherapy and targeted agents, and potentially discovering novel therapeutic targets.

## RESULTS

### Population

2539 sarcoma specimens encompassing 61 bone and STS subtypes were profiled. These included 22 standard histologies, and an “other” (*n* = 454) category, that included sarcoma NOS (*n* = 251) and spindle cell tumor (*n* = 103), as well as 37 sarcoma subtypes that had fewer than 10 cases. The most common histology was leiomyosarcoma (LMS) (*n* = 751) with 401 of these uterine, followed by liposarcoma (*n* = 220) and undifferentiated pleomorphic sarcoma [UPS (formally malignant fibrous histiocytoma) (*n* = 166)]. Of the 77 angiosarcomas, 14 were of breast origin. 862 samples were known to be from a metastatic site. The median age of the population was 53 (range: 1–92). 62% of the cases were from females. Up to 2434 samples were profiled by IHC, 1048 by FISH/CISH, 591 by NGS and 1250 by Sanger sequencing. 530 samples were profiled on all three platforms (IHC, FISH/CISH and either NGS or Sanger sequencing).

### Protein biomarkers

**Figure 1:**
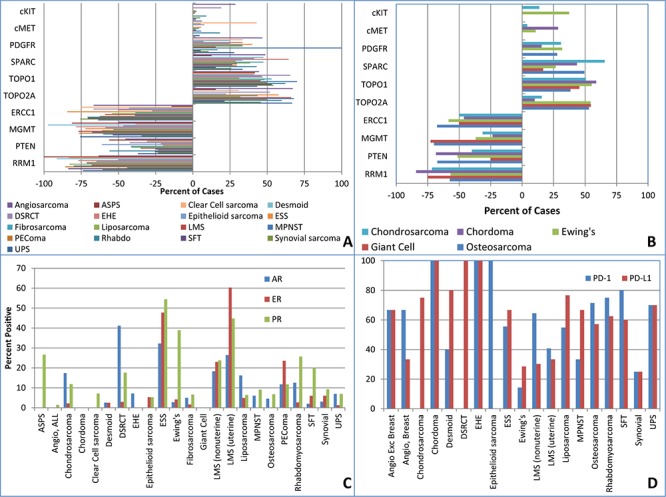
Protein biomarker expression by IHC **A.** Soft tissue sarcomas **B.** Bone sarcomas **C.** Hormone receptors **D.** PD-1, PD-L1. ASPS = alveolar soft part sarcoma, DSRCT = desmoplastic small round cell tumor, EHE = epithelioid hemangioendothelioma ESS = endometrial stromal sarcoma, SFT = solitary fibrous tumor, LMS = leiomyosarcoma, UPS = undifferentiated pleomorphic sarcoma, MPNST = malignant peripheral nerve sheath tumor, PEComa = perivascular epithelioid cell tumor.

TOPO2A overexpression, an anthracycline associated response biomarker, was noted in 52.8% of the sarcomas and in greater than 60% of MPNST, angiosarcoma, LMS, rhabdomyosarcoma and UPS. High expression of TOPO2A has been previously reported at a rate of 50% in STS, when median percentage was chosen as the cutoff to discriminate between high and low expressing tumors, rather than criteria previously described in breast cancer [10, 11]. Overexpression of serum protein acidic and rich in cysteine (SPARC), a biomarker for albumin bound paclitaxel, was seen in 35.9% of the cases, especially in over 60% of epithelioid hemangioendothelioma (EHE) and chondrosarcoma (notably conventional chondrosarcoma), as well as 48.7% of angiosarcomas. A previous study of SPARC expression by IHC, noted a rate of high SPARC staining in 56% of specimens, but given the small sample size (*n* = 27), specific histology correlations could not be made [12]. Low MGMT expression, a temozolomide associated biomarker, was noted in a variety of sarcomas including alveolar soft part sarcoma (21 ASPS), desmoid, EHE, perivascular epithelioid cell tumor (PEComa), endometrial stromal sarcoma (ESS), giant cell tumor, liposarcoma, LMS, malignant peripheral nerve sheath tumor (MPNST), osteosarcoma and UPS. There was low expression of MGMT in 65.3% of the sarcomas overall. Previous studies have only considered nuclear staining positive, and have reported a much lower rate of MGMT loss in LMS and other STS subtypes, therefore validation of a particular method is required to determine the predictive value in STS [13]. PTEN loss was seen in 38.6% of the sarcomas, most commonly in epithelioid sarcoma, chordoma, alveolar rhabdomyosarcoma and osteosarcoma. PTEN loss was only noted in 32.2% of non-uterine LMS and 37.6% of uterine LMS. Previous work on complex genomic sarcomas such as LMS, UPS and MPNST have reported rates of PTEN loss in 29%–44% of the sarcomas, [14, 15] but data on the expression of PTEN in rarer sarcomas is lacking. cKIT overexpression was noted in 28.5% of angiosarcoma, 19% of desmoplastic small round cell tumor (DSRCT) and 37.3% of Ewing's sarcoma. There were 2 cases of sclerosing rhabdomyosarcoma, both of which overexpressed cKIT. This is a rare sarcoma whose treatment paradigm is not yet clearly defined. PDGFRA was overexpressed in 22.1% of the sarcomas, including, 38.5% of angiosarcoma, 33.3% of liposarcoma, 33.3% of fibrosarcoma, 31.8% of Ewing's sarcoma, 30.8% of chondrosarcoma, 27.8% of osteosarcoma, 27.8% of UPS and 18.3% of non-uterine LMS. High PDGFRA expression has been described previously in many of these tumors [16–18]. In a series by Rodrigo and colleagues HER2 protein expression by IHC was negative in all sarcoma samples [10]. HER2 overexpression was noted in only one case in this series, an ESS, confirming that this is not an important pathway in sarcoma (Figure 1a and 1b and Supplementary Table 1).

AR overexpression was seen in 18.3–54.2% of myxoid chondrosarcoma, DSRCT, pleomorphic and well-differentiated liposarcoma, ESS and LMS. ER overexpression was seen in 47.8% of ESS, 23.0% of non-uterine LMS, 60.3% of uterine LMS and 23.5% of PEComas (Figure 1c and Supplementary Table 1). The rates of ER overexpression in uterine LMS appear similar to previous studies [19, 20]. This data also confirms the rate of ER positivity in non-uterine LMS at approximately 20% with a much larger number of cases [21].

Overall, approximately 50% of the sarcomas expressed PD-L1 by IHC and presented with PD-1+ TILs, similar to other series [22]. Sarcomas with sufficient number of cases and overexpression of these proteins included LMS, chondrosarcoma, liposarcoma and UPS. Only 4 cases of synovial sarcoma were available for testing, 1 of which had simultaneous overexpression of PD-L1 and presence of PD-1+ TILs (Figure 1d and Supplementary Table 2). This rate is lower than published literature [22].

### FISH/CISH

The most commonly amplified gene was *EGFR* at a rate of 16.9% overall. This was especially noted in histologies such as DSRCT, LMS, MPNST, osteosarcoma and UPS at a rate of 20% or higher. Smaller sarcoma series have suggested a lower rate of amplification overall (3.5%), most commonly in UPS [23]. In series focusing on MPNST specifically, the rate of *EGFR* gene amplification by FISH was 28–37% [24, 25]. None of the synovial sarcomas in our series demonstrated genomic amplification of *EGFR*, consistent with previous work [26]. Amplification of the *ALK, cMYC, PIK3CA and TOPO2A* genes were relatively uncommon events. *cMET* was amplified in 5–6% of osteosarcoma and synovial sarcomas. *HER2* was amplified in 5.6% of MPNST (Table 1).

**Table 1:** FISH/CISH by histology

Histology*		Gene**		
		ISH cMET	ISH EGFR	ISH HER2
All (*n* = 2539)	Total Positive	20	181	9
	Total Cases	761	1072	925
	% Positive	2.6	16.9	1.0
Angiosarcoma (*n* = 77)	Total Positive	0	1	0
	Total Cases	26	34	33
	% Positive	0.0	2.9	0.0
Chondrosarcoma (*n* = 97)	Total Positive	1	2	1
	Total Cases	32	24	42
	% Positive	3.1	8.3	2.4
Clear cell sarcoma (*n* = 16)	Total Positive	0	1	0
	Total Cases	4	8	4
	% Positive	0.0	12.5	0.0
DSRCT (*n* = 8)	Total Positive	0	2	0
	Total Cases	11	10	15
	% Positive	0.0	20.0	0.0
ESS (*n* = 91)	Total Positives	0	4	1
	Total Cases	33	50	31
	% Positive	0.0	8.0	3.2
Ewing's sarcoma (*n* = 83)	Total Positive	0	2	0
	Total Cases	23	30	27
	% Positive	0.0	6.7	0.0
Fibrosarcoma (*n* = 63)	Total Positive	0	5	0
	Total Cases	21	33	21
	% Positive	0.0	15.2	0.0
Giant cell tumor (*n* = 13)	Total Positive	0	2	0
	Total Cases	3	7	4
	% Positive	0.0	28.6	0.0
Liposarcoma (*n* = 220)	Total Positive	3	14	0
	Total Cases	62	80	93
	% Positive	4.8	17.5	0.0
Myxoid (*n* = 46)	Total Positive	1	0	0
	Total Cases	14	15	18
	% Positive	7.1	0.0	0.0
Dedifferentiated (*n* = 77)	Total Positive	0	1	0
	Total Cases	24	28	41
	% Positive	0.0	3.6	0.0
Well-differentiated (*n* = 31)	Total Positive	0	5	0
	Total Cases	11	12	13
	% Positive	0.0	41.7	0.0
Pleomorphic (*n* = 30)	Total Positive	2	5	0
	Total Cases	7	8	10
	% Positive	28.6	62.5	0.0
Other/Unknown (*n* = 36)	Total Positive	0	3	0
	Total Cases	6	17	11
	% Positive	0.0	17.6	0.0
LMS (nonuterine) (*n* = 350)	Total Positive	4	27	3
	Total Cases	112	128	137
	% Positive	3.6	21.1	2.2
LMS (uterine) (*n* = 401)	Total Positive	4	43	1
	Total Cases	96	220	106
	% Positive	4.2	19.5	0.9
MPNST (*n* = 36)	Total Positive	0	4	1
	Total Cases	13	14	18
	% Positive	0.0	28.6	5.6
Osteosarcoma (*n* = 95)	Total Positive	1	9	0
	Total Cases	18	46	24
	% Positive	5.6	19.6	0.0
PEComa (*n* = 17)	Total Positive	0	2	0
	Total Cases	9	7	9
	% Positive	0.0	28.6	0.0
Rhabdomyosarcoma (*n* = 82)	Total Positive	0	4	1
	Total Cases	27	26	36
	% Positive	0.0	15.4	2.8
Alveolar (*n* = 18)	Total Positive	0	1	0
	Total Cases	1	4	3
	% Positive	0.0	25.0	0.0
Embryonal (*n* = 19)	Total Positive	0	0	0
	Total Cases	7	8	6
	% Positive	0.0	0.0	0.0
Pleomorphic (*n* = 9)	Total Positive	0	0	0
	Total Cases	5	1	7
	% Positive	0.0	0.0	0.0
Other/Unknown (*n* = 36)	Total Positive	0	3	1
	Total Cases	14	12	20
	% Positive	0.0	25.0	5.0
SFT (*n* = 56)	Total Positive	0	1	0
	Total Cases	11	28	18
	% Positive	0.0	3.6	0.0
Synovial sarcoma (*n* = 70)	Total Positive	1	0	0
	Total Cases	19	31	21
	% Positive	5.3	0.0	0.0
UPS (*n* = 166)	Total Positive	2	30	0
	Total Cases	54	76	66
	% Positive	3.7	39.5	0.0
Other (*n* = 454)	Total Positive	4	28	1
	Total Cases	156	182	177
	% Positive	2.6	15.4	0.6

DSRCT = desmoplastic small round cell tumor, ESS = endometrial stromal sarcoma, LMS = leiomyosarcoma, UPS/MFH = undifferentiated pleomorphic sarcoma/malignant fibrous histiocytoma, MPNST = malignant peripheral nerve sheath tumor, PEComa = perivascular epithelioid cell tumor, SFT = solitary fibrous tumor,

* Histologies with no amplification of genes tested: alveolar soft part sarcoma, chordoma, desmoid, epithelioid hemangioendothelioma, epithelioid sarcoma.

** PIK3CA – 3/9 cases amplified( LMS, osteosarcoma, other); TOPO2A – 2/118 cases amplified(LMS, other); cMYC – 1/18 cases amplified(osteosarcoma); ALK – 1/65 cases amplified (LMS).

### DNA sequencing

**Figure 2:**
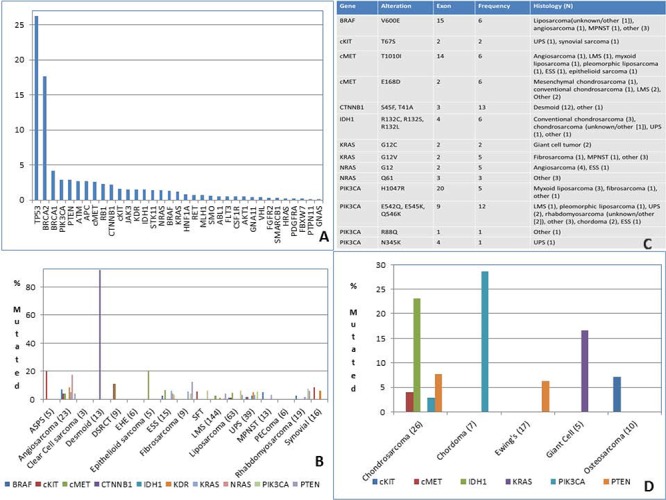
DNA sequencing in sarcoma **A.** Most frequent mutation (%) **B.** Mutation type **C.** Mutations in STS **D.** Mutations in bone sarcoma. ASPS = alveolar soft part sarcoma, DSRCT = desmoplastic small round cell tumor, ESS = endometrial stromal sarcoma, SFT = solitary fibrous tumor, LMS = leiomyosarcoma, UPS = undifferentiated pleomorphic sarcoma, MPNST = malignant peripheral nerve sheath tumor, PEComa = perivascular epithelioid cell tumor.

591 samples were profiled by NGS and 1250 by Sanger sequencing. 47% of the samples had an identifiable mutation in 35 of the 47 genes analyzed. The most commonly mutated genes overall were *TP53* (26.3%) and *BRCA2* (17.6%). Histologies carrying mutations at a frequency of ≥ 5% included: angiosarcoma (*APC, BRAF, GNA11, HRAS KDR, KRAS, NRAS*), chondrosarcoma [*IDH1* (conventional and unknown/other), *PTEN* (myxoid), *cMET* (conventional and mesenchymal)], desmoid (*APC, CTNNB1, STK11*), ESS (*AKT1, cMET, FGFR2, GNAS, KRAS, RET, SMO*), Ewing's sarcoma (*APC, ATM, HNF1A, PTEN*), fibrosarcoma (*KRAS*), giant cell tumor (*KRAS*), myxoid liposarcoma (*AKT1, ATM, cMET, JAK3, PIK3CA, PTEN*), dedifferentiated liposarcoma (*HNF1*), well-differentiated liposarcoma (*cMET*), LMS (*BRCA2, RB1*), MPNST (*BRAF* V600E), osteosarcoma (*cKIT, FLT3*), rhabdomyosarcoma [*FLT3* (pleomorphic), *PIK3CA* (uknown/other), *PTEN* (unknown/other), *PTPN11* (alveolar), *SMARCB1* (unknown/other)], solitary fibrous tumor (*cKIT, PDGFRA, PIK3CA, STK11*), synovial sarcoma (*ABL1, ATM, BRAF, cKIT, KDR, MLH1*) and UPS (*KDR, PIK3CA*). *BRCA2* mutations were seen in 17% of LMS, both uterine and non-uterine. *PTEN* and *RB1* mutations were noted exclusively in non-uterine LMS and not in those of uterine origin. *NRAS* mutations were detected in 20% of non-breast angiosarcomas, and were not found in those of breast origin. The *PIK3CA* mutations noted in liposarcoma (5 cases) were found in 4 myxoid liposarcomas and in 1 high grade pleomporphic liposarcoma. No *EGFR* mutations were detected in our series (Figure 2a–d and Supplementary Table 3). One case of MPNST carried a G12V *KRAS* mutation. We detected *BRAF, PTEN, p53* and *NRAS* mutations in angiosarcoma specimens, not previously described in the literature [4].

### Biomarker associations

There was relatively low concordance across platforms for individual genes or proteins (Table 2). For example, cKIT overexpression by IHC was infrequently associated with *cKIT* mutations. This is in contrast to the scenario in GIST, where more than 80% of cases carry an activating mutation in the KIT gene [27]. In our series, overexpression of TOPO2A by IHC was not associated with *TOPO2A* gene amplification, similar to some series in breast cancer and in contrast to others [28, 29]. We also examined the association of TP53 or PIK3CA mutations with other alterations (Table 3 and Table 4). We noted that 85.8% of samples demonstrated both TOPO2A expression by IHC and *TP53* mutation (*P* value = 0.0001). Three patient tumor samples had both a PIK3CA mutation and PTEN loss by IHC (myxoid liposarcoma, rhabdomyosarcoma and sarcoma, NOS). Two patient samples had *EGFR* amplification and *KRAS* mutation (1 MFH and 1 ESS).

**Table 2:** Concordance across platforms

	N positive concordance/N	IHC+(%)	FISH amplified (%)	Mutation (%)
cKIT	0/23	65/1441 (4.5)	NA	12/749 (1.6) All VUS*
cMET	1/751 (IHC/ISH) 0/442 (ISH/NGS) 1/567 (IHC/NGS)	43/970 (4.4)	20/761 (2.6)	15/588 (2.6) All VUS*
EGFR	2/45 (IHC/ISH) 0/16 (ISH/NGS) 0/11 (IHC/NGS)	80/217 (36.9)	181/1072 (16.9)	0/608 (0)
HER2	0/910 (IHC/ISH) 0/518 (ISH/NGS) 0/555 (IHC/NGS	1/2409 (0.04) *Case with HER2 overexpression was not tested on other platforms	9/925 (1.0)	0/573 (0)
PDGFR	NA	135/610 (22.1)	NA	1/581 (0)
PTEN	8/539 (IHC/NGS)	Loss 910/2358 (38.6)	NA	16/557(15 cases) (3.2) *8/15 cases with PTEN mutation had PTEN loss *7/15 cases with mutation were VUS without PTEN loss
TOPO2A	0/36(IHC/FISH)	1117/2114 (52.8)	2/118 (1.7)	NA

* VUS = variant of unknown significance

**Table 3:** Biomarker associations

	PTEN Loss IHC	TOPO2 IHC+	PTEN MT	cMET MT	IDH MT	CTNNB1 MT	APC MT	KRAS MT
TP53wt	56/414 (13.5%)	226/396 (57.1%)	9/407 (2.2%)	8/427 (1.9%)	5/430 (1.2%)	13/430 (3.0%)	11/429 (2.6%)	7/429 (1.6%)
TP53 mutated	22/135 (16.3%)	115/134 (85.8%)	5/127 (3.9%)	6/136 (4.4%)	4/136 (2.9%)	0/136 (0)	5/135 (3.7%)	1/135 (0.7%)
*P* value	0.48	0.0001	0.34	0.11	0.23	0.045	0.55	0.69

**Table 4:** Biomarker associations

	TP53 MT (%)	PTEN Loss IHC (%)	TOPO2 IHC+ (%)	PTEN MT (%)
PIK3CA mutated	5/20 (25.0%)	3/22 (13.6%)	14/20 (70.0%)	2/18 (11.1%)
PIK3CA WT	140/533 (26.3%)	143/781 (18.3%)	367/567 (64.7%)	13/529 (2.5%)
*P* value	1.0	0.78	0.81	0.08

## DISCUSSION

This data set represents the largest number of sarcoma cases molecularly profiled to date. We observed that TOPO2A was overexpressed in approximately 50% of sarcomas, without associated gene amplification, most commonly in the angiosarcomas, LMS, MPNST, rhabdomyosarcoma and UPS. Indeed, amplification of the *TOPO2A* gene has not reliably predicted increased protein levels of TOPO2A in other cancers [11, 29, 30].

Anthracylines have served as a cornerstone for the treatment of sarcomas for decades. Response rates to single agent doxorubicin range from 9% to 27% [31, 32], with clinical benefit in approximately 60% of patients [33]. The mechanisms related to the lack of benefit seen in some patients are not entirely clear. TOPO2A is an important target for anthracyclines and its inhibition leads to double-strand DNA breaks and cell death. In breast cancer, TOPO2A protein expression by IHC has been correlated with response to anthracyclines [34], however this has been confounded by the co-expression of HER2. In sarcoma, a retrospective study of TOPO2A expression in 78 patients who underwent neoadjuvant therapy with an anthracycline, found that histologic response was positively correlated with high expression of TOPO2A [10]. This finding requires prospective validation. Although previous work has suggested overexpression of HER2 in approximately 50% of synovial sarcomas [26], none of the sarcoma specimens in this series co-expressed HER2. These findings also suggest that TOPO2A protein expression is not controlled by gene copy number, but through RNA or post-translational mechanisms. Our analysis also found an association between TOPO2A overexpression by IHC and TP53 mutation status. Indeed, in some studies, breast tumors containing *TP53* mutations are exquisitely sensitive to anthracycline based therapy [35]. It has been postulated and shown in *TP53* wild type xenograft models that upon treatment with doxorubicin, there is induction of a senescent phenotype leading to cell cycle arrest and subsequent resistance to treatment [36, 37]. This mutational status could therefore serve as a biomarker for sensitivity to anthracyclines in sarcoma and requires further study.

SPARC is a serum albumin-binding glycoprotein secreted by endothelial cells. It is hypothesized that tumoral SPARC could serve as a biomarker for sensitivity to the albumin-bound nanopartical of paclitaxel NAB-paclitaxel. In pancreatic cancer, SPARC overexpression was correlated with response rate to nab-paclitaxel and prolonged progression free survival [38]. Clinically, angiosarcomas and EHE are essentially the only sarcoma subtypes where single agent taxanes are of benefit [39]. Though, SPARC expression has been assessed in sarcoma specimens, only 1 case of angiosarcoma has previously been evaluated and was found to have high expression of SPARC [12]. In our study, interestingly, SPARC was overexpressed in angiosarcoma, chondrosarcoma and EHE. A clinical trial of nab-paclitaxel in a wide variety of bone and soft tissue sarcomas did not meet its primary endpoint. However, this study did not assess for SPARC status or include patients with angiosarcoma or EHE [40]. MGMT gene silencing through promotor methylation confers sensitivity to the alkylating agents in glioblastoma [41]. Previous work has suggested that 20% of STS have MGMT loss by IHC and 8% have both promoter methylation and negative IHC nuclear staining. Response to temozolomide has been noted in a patient with a resistant undifferentiated high grade sarcoma whose tumor demonstrated MGMT promoter methylation and protein loss by IHC [42].

Targeted therapy has improved outcomes of patients with solid tumors such as lung and kidney cancer. Pazopanib remains the only FDA approved targeted therapy in STS, as other trials of novel agents have largely been unsuccessful when tested in a broad- range of sarcomas. Biomarkers predicting response would therefore be of utility. The PI3-kinase pathway is of great interest in sarcoma and cancer in general. Previous studies have shown *PTEN* partial genomic loss and loss of protein expression in LMS. Gibault and colleagues showed that partial loss of *PTEN* gene by array-comparative genomic hybridization (a-CGH) occurs in 39% of sarcomas with complex genomics and that this may be sufficient for a pathologic phenotype [14]. On the other hand, PTEN promoter methylation appears to be an uncommon event and may not play a major role in down-regulation of PTEN expression [14, 15]. In our study PTEN loss was most notable in the chordomas, epithelioid sarcomas, alveolar rhabdomyosarcomas and osteosarcomas. Though loss was noted in the LMS cohort, this was only seen in about 35% of the cases. Disappointingly, targeting this pathway with mTOR inhibitors has been unsuccessful clinically [43], possibly owing to the fact that preselection of tumors with alterations in the PI3-kinase pathway was not required for trial enrollment. Overall, the rate of *PIK3CA* and *PTEN* mutations was low in our series. *PTEN* mutations were most commonly seen in chondrosarcomas (7.7%), myxoid liposarcomas (16.7%), Ewing's sarcoma and rhabdomyosarcoma (6.3%). Previous work has demonstrated *PIK3CA* mutations in 14–18% of myxoid and round cell liposarcoma [3, 5]. In our series, *PIK3CA* mutations were most commonly seen in myxoid liposarcoma (23.5%), as well as in rhabdomyosarcoma (7.4%), solitary fibrous tumor (6.3%) and UPS (5.5%). We had 1 case of myxoid liposarcoma with both PTEN loss by IHC and *PIK3CA* mutation, not previously described in the literature [3]. We also detected pathogenic *PTEN* mutations in angiosarcoma specimens, which have not previously been described in the literature [4]. Finally, our data suggests the co-existence of *PTEN* and *TP53* mutations, as well as *TP53* mutations and PTEN loss in sarcoma specimens. Indeed, it is known that there is considerable crosstalk between these two tumor suppressor genes [44, 45]. The prognostic effect of this phenomenon is not known in sarcoma; therefore validation against phase III clinical data is needed. Our data also confirmed the rate of ER positivity in LMS. A phase II study of the aromatase inhibitor letrozole in these patients has demonstrated clinical benefit [46]. On the other hand, though PDGFRA was overexpressed in many tumors consistent with previous work [16–18], phase II studies of imatinib in PDGFR- positive sarcomas failed to show benefit [18, 47].

Newer immunotherapies have demonstrated success in melanoma, kidney cancers and lung cancers, but have not yet been evaluated in sarcoma [48]. Currently of most interest are the PD-1 and PD-L1 inhibitors. Previous work has shown that both PD-1 and PD-L1 positivity were independent prognostic indicators for OS and EFS in sarcoma [22]. PD-1 positive tumor-infiltrating lymphocytes and PD-L1 expression in tumor cells were seen in 65% and 58% of STS cases respectively. Uncertainty remains as to the effectiveness of PD-L1 expression in tumor as a biomarker for sensitivity to the checkpoint inhibitors as patients with low or no expression of PD-L1 may still have a response and/or survival benefit [49]. PD-L1 expression was noted in the majority of chondrosarcomas, LMS, UPS and liposarcoma in our series.

Protein expression is a semi-quantitative, inclusive end-point. Changes in protein level may be a result of mutations, increased gene copy number, changes in gene expression, or biochemical responses within the cell to environmental factors. In other solid tumors, there is concordance between protein expression by IHC and mutation or gene amplification as in the cases of cKIT and HER2 respectively [50, 51]. However, discordance has been documented across solid tumors for cMET, TOP2A, and PTEN [30], proposed to be due to downstream modulation of protein expression levels. There was relatively low concordance across platforms in our series, suggesting that protein levels of cKIT, cMET and TOPO2A may be regulated at the RNA or post translational level. On the other hand, IHC for certain proteins such as EGFR, is known to be unreliable, owing the antibodies and scoring system used [23]. In fact, a prior clinical trial of gefitinib in synovial sarcomas with enrollment limited to patients overexpressing EGFR by IHC was without responses to this agent. Twenty-one percent of patients did have stable disease [52]. In addition, no *EGFR* mutations were detected in our sarcomas, consistent with other series [24]. Our data set suggests that *EGFR* gene amplification could be used instead of IHC as a biomarker for sensitivity to the EGFR inhibitors. This gene amplification was most notable in DSRCT, LMS, MPNST, osteosarcoma and UPS.

There are several limitations to this analysis. Though all specimens underwent pathology review to confirm a diagnosis of sarcoma, the specific subtype was determined by the submitting institution's pathologist. This is problematic for non-specific diagnoses such as fibrosarcoma, which may in fact represent a heterogenous group of tumors. The lack of concordance across platforms observed in this series is of interest. This may be attributed, as in the case of EGFR, to the unreliability of IHC. In this case, it seems that copy number analysis may be more useful. On the other hand, the lack of concordance between *cKIT* activating mutations and increased protein expression suggests an inadequacy in the methodology used to detect these genetic alterations. In fact, there was a low frequency of actionable mutations detected in this series overall, suggesting that Hotspot analysis has limited scope in sarcoma and techniques such as whole exome sequencing should be explored. Despite these limitations in technique, there were a few noteworthy observations, including alteration of the RAF/RAS/MEK/ERK pathway in angiosarcoma. Finally, this analysis does not include details on treatment history and/or patient outcomes which would be useful as a first step in validation of these potential biomarkers.

These data represent a large analysis of molecular alterations by sarcoma subtype. Although there are limitations of this analysis, this data catalogue provides the groundwork for future clinical trials in sarcoma and highlights the need for the sarcoma community to prospectively validate tumor biomarkers in our clinical trials.

## MATERIALS AND METHODS

Data from sarcoma specimens profiled on at least one platform by Caris Life Sciences from January 11, 2006 through July 31, 2014 were included. Formalin fixed paraffin-embedded (FFPE) sarcoma samples were sent for analysis from treating physicians around the world (59 countries). The specific sarcoma histology was extracted from paperwork submitted by the treating physician. GIST was excluded, to limit the analysis to sarcomas without known therapeutic targets. Tumors were initially verified by a board certified pathologist for sufficient tumor presence and to confirm the diagnosis of sarcoma. Any sample reviewed and determined not to be a sarcoma was excluded from this cohort. Samples were subsequently analyzed using one or more of the profiling platforms as described below. Biomarkers for analysis were selected based on their potential to be targeted therapeutically and/or based on clinical evidence of a utility in other solid tumors.

### Immunohistochemistry (IHC)

Protein expression was determined by IHC analysis, using commercially available detection kits and automated staining techniques (Benchmark XT, Ventana, and AutostainerLink 48, Dako) [53]. Antibodies used included: androgen receptor (AR), topoisomerases 1 and 2 (TOPO1, TOPO2A) (Leica Biosystems); ER, PR, cMET, HER2 (Ventana); cKIT, epidermal growth factor receptor (EGFR), phosophatase and tensin homolog (PTEN) (Dako), O(6)-methylguanine-methyltransferase (MGMT), P-glycoprotein (PGP) (Invitrogen); transducin-like enhancer of split 3 (TLE3, Santa Cruz); ribonucleotide reductase M1 (RRM1, Protein Tech); SPARC (monoclonal, R&D Systems; polyclonal, Exalpha), tubulin beta-3 chain (TUBB3) (Covance), Excision Repair Cross-Complementation Group 1 (ERCC1, (Abcam), platelet derived growth factor receptor alpha (PDGFRA, Thermo), Programmed cell death protein 1 (PD-1) and Programmed death-ligand 1 (PD-L1) (BD Pharmingen and R&D Systems). IHC thresholds previously validated in other cancers were used, as cutoffs are not established in sarcoma (Appendix 1).

**Appendix 1:** IHC Thresholds

Protein	Threshold for Overexpression (Intensity and %expression in tumor)
AR	≥ 1+ and ≥ 10%
cKIT	≥ 2+ and ≥ 30%
cMET	≥ 2+ and ≥ 50%
EGFR	2+ and = 10% or 1+ and < 10%; current = H-score, < 200 or ≥ 200
ER	≥ 1+ and ≥ 10%
PR	≥ 1+ and ≥ 10%
HER2	≥ 3+ and > 10%
PD-1	≥ 1+ (TIL Count/High power field with 40x objective)
PD-L1	≥ 2+ and ≥ 5%
PDGFRA	≥ 2+ and 30%
PGP	≥ 1+ and ≥ 10%
SPARC	≥ 2+ and ≥ 30%
TLE3	≥ 2+ and ≥ 30%
TOPO1	≥ 2+ and ≥ 30%
TOP2A	≥ 1+ and ≥ 10%
TUBB3	≥ 2+ and ≥ 30%
**Protein**	**Threshold for Loss (Intensity and %expression in tumor)**
ERCC1	< 2+ or ≤ 3+ and < 10% or = 2+ and < 50%
MGMT	= 0+ or ≤ 35%
PTEN	= 0+ or ≤ 50%
RRM1	< 2+ or < 50%

### *In situ* hybridization

Fluorescent *in-situ* hybridization (FISH) was used for evaluation of the *HER-2/neu* [HER-2/CEP17 probe; HER-2/CEP17 ratio > 2.2 was considered amplified], *EGFR* [EGFR/CEP7 probe EGFR/CEP7 ratio ≥ 2, or ≥ 15 EGFR copies per cell in ≥ 10% of analyzed cells was considered amplified], *TOP2A* [TOP2/CEP17 probe; TOP2A/CEP17 ratio ≥ 2.0 was considered amplified], *cMET* [cMET/CEP7 probe; cMET/CEP7 ratio ≥ 5 was considered amplified], *ALK* [Break Apart Probe; ALK rearrangements, either inversion or translocation of the ALK gene at 2p23, were identified by separation of the fusion signals into one red and one green signal], *cMYC* [cMYC/CEP8 probe; cMYC/CEP8 ratio ≥ 2 was considered amplified], and *PIK3CA* [PIK3CA/CEP3 probe; PIK3CA/CEP3 ≥ 3 was considered amplified] (Abbott Molecular/Vysis). *HER-2/neu* and *cMET* status were more recently evaluated by chromogenic *in-situ* hybridization (INFORM HER-2 Dual ISH DNA Probe Cocktail; commercially available cMET and chromosome 7 DIG probe; Ventana), and used the same scoring system as for FISH.

### Mutational analysis

#### Next-Generation Sequencing (NGS)

Direct sequence analysis was performed on genomic DNA isolated from FFPE tumor samples using the Illumina MiSeq platform. Average sequencing depth was > 1000X. Specific regions of 47 genes were amplified using the Illumina TruSeq Amplicon Cancer Hotspot panel.

#### Sanger sequencing

Prior to the availability of CLIA certified NGS, mutation analysis by Sanger sequencing included selected regions of *BRAF, KRAS, cKIT, EGFR,* and *PIK3CA* genes and was performed by using M13-linked PCR primers designed to amplify targeted sequences. PCR products were bi-directionally sequenced using the BigDye Terminator v1.1 chemistry, analyzed using the 3730 DNA Analyzer (Applied Biosystems). Sequence traces were analyzed using Mutation Surveyor software v3.25 (Soft Genetics).

### Statistical analysis

The patient population and profiling data were characterized using standard descriptive statistics. When comparing data across the subtypes, groups with less than 10 cases were not considered. For chemotherapy protein biomarkers, overexpression or loss in at least 60% of samples in a particular subtype were considered clinically significant (mean selected as cutoff). For targeted therapy protein biomarkers, overexpression in at least 17.5% of cases was considered clinically relevant (mean selected as cutoff). Concordance across platforms was determined using Cohen's Kappa.

## SUPPLEMENTARY TABLES


