# Supplementary material for: Multi-platform profiling of over 2000 sarcomas: Identification of biomarkers and novel therapeutic targets
Source: Oncotarget. 2015 Mar 26;6(14):12234–47. doi: 10.18632/oncotarget.3498 (PMC4494935; doi:10.18632/oncotarget.3498)
Supplement: Supplementary file 1 [file oncotarget-06-12234-s001.pdf]

SUPPLEMENTARY TABLES

Supplementary Table S1. IHC by histology

Supplementary Table S2. PD-1, PD-L1 expression by histology

Histology		IHC PD-1	IHC PD-L1
All (*n* = 2539)	Total Positive	120	126
	Total Cases	219	221
	% Positive	54.8	57.0
Angiosarcoma (*n* = 77)	Total Positive	4	3
	Total Cases	6	6
	% Positive	66.7	50.0
Chondrosarcoma (*n* = 97)	Total Positive	0	12
	Total Cases	16	16
	% Positive	0.0	75.0
Chordoma (*n* = 24)	Total Positive	1	1
	Total Cases	1	1
	% Positive	100.0	100.0
Desmoid (*n* = 43)	Total Positive	2	4
	Total Cases	5	5
	% Positive	40.0	80.0
DSRCT (*n* = 36)	Total Positive	0	1
	Total Cases	0	1
	% Positive	0.0	100.0
EHE (*n* = 14)	Total Positive	1	1
	Total Cases	1	1
	% Positive	100.0	100.0
Epithelioid sarcoma (*n* = 19)	Total Positive	1	0
	Total Cases	1	1
	% Positive	100.0	0.0
ESS (*n* = 91)	Total Positive	5	6
	Total Cases	9	9
	% Positive	55.6	66.7
Ewing's sarcoma (*n* = 83)	Total Positive	1	2
	Total Cases	7	7
	% Positive	14.3	28.6
Leiomyosarcoma (*n* = 751)	Total Positive	31	19
	Total Cases	58	60
	% Positive	53.4	31.7

(*Continued*)

Histology		IHC PD-1	IHC PD-L1
Liposarcoma (*n* = 220)	Total Positive	17	23
	Total Cases	31	30
	% Positive	54.8	76.7
Myxoid (*n* = 46)	Total Positive	2	6
	Total Cases	6	6
	% Positive	33.3	100.0
Dedifferentiated (*n* = 77)	Total Positive	7	9
	Total Cases	12	11
	% Positive	58.3	81.8
Well-differentiated (*n* = 31)	Total Positive	2	3
	Total Cases	3	3
	% Positive	66.7	100.0
Pleomorphic (*n* = 30)	Total Positive	3	2
	Total Cases	4	4
	% Positive	75.0	50.0
MPNST (*n* = 36)	Total Positive	1	2
	Total Cases	3	3
	% Positive	33.3	66.7
Osteosarcoma (*n* = 95)	Total Positive	5	4
	Total Cases	7	7
	% Positive	71.4	57.1
Rhabdomyosarcoma (*n* = 82)	Total Positive	6	5
	Total Cases	8	8
	% Positive	75.0	62.5
Embryonal (*n* = 9)	Total Positive	0	0
	Total Cases	1	1
	% Positive	0.0	0.0
Pleomorphic (*n* = 19)	Total Positive	2	1
	Total Cases	2	2
	% Positive	100.0	50.0
Solitary fibrous tumor (*n* = 56)	Total Positive	4	3
	Total Cases	5	5
	% Positive	80.0	60.0
Synovial sarcoma (*n* = 70)	Total Positive	1	1
	Total Cases	4	4
	% Positive	25.0	25.0

(*Continued*)

Histology		IHC PD-1	IHC PD-L1
UPS (n = 166)	Total Positive	7	7
	Total Cases	10	10
	% Positive	70.0	70.0
Other (n = 454)	Total Positive	33	32
	Total Cases	47	47
	% Positive	70.2	68.1

DSRCT = desmoplastic small round cell tumor, EHE = epithelioid hemangioendothelioma ESS = endometrial stromal sarcoma, LMS = leiomyosarcoma, UPS = undifferentiated pleomorphic sarcoma, MPNST = malignant peripheral nerve sheath tumor, SFT = solitary fibrous tumor.

Supplementary Table S3. DNA sequencing by histology
